# P-2226. Clinical Validation and Performance of Molecular Syphilis Diagnosis by Loop-Mediated Isothermal Amplification

**DOI:** 10.1093/ofid/ofae631.2380

**Published:** 2025-01-29

**Authors:** Hayden Z Smith, Yijun Sim, Jon Kingma, Ichih Wang-McGuire, Lauren Tantalo, Khalil Deveaux, Austin Haynes, Greg Levin, Ka-Wing Sullivan, Regina Kwon, Brad Cookson, Lorenzo Giacani, Stephen J Salipante, Joshua Lieberman

**Affiliations:** University of Washington, Seattle, Washington; University of Washington, Seattle, Washington; University of Washington, Seattle, Washington; University of Washington, Seattle, Washington; University of Washington, Seattle, Washington; University of Washington, Seattle, Washington; University of Washington, Seattle, Washington; University of Washington, Seattle, Washington; University of Washington, Seattle, Washington; University of Washington, Seattle, Washington; University of Washington, Seattle, Washington; University of Washington, Seattle, Washington; University of Washington, Seattle, Washington; University of Washington, Seattle, Washington

## Abstract

**Background:**

Syphilis cases continue to increase rapidly in the US. Molecular detection of the pathogen, *Treponema pallidum* (TP) may speed turnaround time (TAT) and resolve ambiguous results from current diagnostics methods, which require stepwise serologic testing. Here we describe validation and clinical performance of a loop-mediated isothermal amplification (LAMP) assay for TP.

**Methods:**

Candidate LAMP primers targeting the 23S rRNA locus of TP were evaluated and the best performing set was used for clinical validation per Clinical Laboratory Standards Institute guidelines. We determined the sensitivity, specificity, precision, accuracy, and reproducibility of the TP-LAMP assay in fresh and formalin-fixed tissue, body fluids, and whole blood using contrived specimens spiked with intact treponemes and residual clinical specimens. For whole blood, automated nucleic acid extraction was performed from 1mL ETDA-anticoagulated sample.

**Results:**

The 95% limit of detection was determined to be 7-10 genomes/reaction by testing remnant DNA eluates spiked with TP gDNA. Assay specificity was evaluated by testing synthetic DNA targets representing non-cultivable targets and residual clinical samples containing DNA from 114 clinically relevant microbes, including non-TP spirochetes, bacteria, fungi, and viruses. TP-LAMP detected 100% of whole blood samples spiked with 120-125 organisms/mL; 88% of samples with 85 organisms/mL; and 80% of samples spiked with 60 organisms/mL.

From 09/2022 - 07/2023 TP-LAMP had 110 results in non-blood samples and the mean TAT was 4.7 days. Orthogonal diagnostic results were available for a subset of 34 samples. In this group the assay was 100% sensitive and specific with 7 positive tests, comprising 2 cases of primary and 5 cases of secondary syphilis. Three tissue specimens with negative TP-LAMP results had false-positive immunohistochemical TP stains due to cross-reactivity with commensal treponemes.

**Conclusion:**

This assay represents a novel, clinically available method for sensitive and specific syphilis diagnosis. Additionally, the assay can resolve ambiguous findings in tissue. Applications in blood may aid in diagnosis of early or latent disease and resolution of unclear serologic test results.

**Disclosures:**

Stephen J. Salipante, M.D. Ph.D., Anavasi: Grant/Research Support Joshua Lieberman, MD, PhD, Anavasi: Grant/Research Support

